# Determination of Resistant Starch Assimilating Bacteria in Fecal Samples of Mice by *In vitro* RNA-Based Stable Isotope Probing

**DOI:** 10.3389/fmicb.2017.01331

**Published:** 2017-07-24

**Authors:** Elena Herrmann, Wayne Young, Douglas Rosendale, Ralf Conrad, Christian U. Riedel, Markus Egert

**Affiliations:** ^1^Faculty of Medical & Life Sciences, Institute of Precision Medicine, Furtwangen University Villingen-Schwenningen, Germany; ^2^AgResearch Ltd., Food Nutrition and Health Team, Grasslands Research Centre Palmerston North, New Zealand; ^3^The New Zealand Institute for Plant & Food Research Ltd. Palmerston North, New Zealand; ^4^Department of Biogeochemistry, Max Planck Institute for Terrestrial Microbiology Marburg, Germany; ^5^Institute of Microbiology and Biotechnology, University of Ulm Ulm, Germany

**Keywords:** resistant starch, gut microbiota, RNA-SIP, *Bacteroidetes*, *Prevotellaceae*, *Ruminococcaceae*, HPLC-IRMS

## Abstract

The impact of the intestinal microbiota on human health is becoming increasingly appreciated in recent years. In consequence, and fueled by major technological advances, the composition of the intestinal microbiota in health and disease has been intensively studied by high throughput sequencing approaches. Observations linking dysbiosis of the intestinal microbiota with a number of serious medical conditions including chronic inflammatory disorders and allergic diseases suggest that restoration of the composition and activity of the intestinal microbiota may be a treatment option at least for some of these diseases. One possibility to shape the intestinal microbiota is the administration of prebiotic carbohydrates such as resistant starch (RS). In the present study, we aim at establishing RNA-based stable isotope probing (RNA-SIP) to identify bacterial populations that are involved in the assimilation of RS using anaerobic *in vitro* fermentation of murine fecal material with stable [U^13^C] isotope-labeled potato starch. Total RNA from these incubations was extracted, processed by gradient ultracentrifugation and fractionated by density. 16S rRNA gene sequences were amplified from reverse transcribed RNA of high and low density fractions suspected to contain labeled and unlabeled RNA, respectively. Phylogenetic analysis of the obtained sequences revealed a distinct subset of the intestinal microbiota involved in starch metabolism. The results suggest *Bacteroidetes*, in particular genera affiliated with *Prevotellaceae*, as well as members of the *Ruminococcacea* family to be primary assimilators of resistant starch due to a significantly higher relative abundance in higher density fractions in RNA samples isolated after 2 h of incubation. Using high performance liquid chromatography coupled to isotope ratio mass spectrometry (HPLC-IRMS) analysis, some stable isotope label was recovered from acetate, propionate and butyrate. Here, we demonstrate the suitability of RNA-SIP to link specific groups of microorganisms with fermentation of a specific substrate. The application of RNA-SIP in future *in vivo* studies will help to better understand the mechanisms behind functionality of a prebiotic carbohydrate and its impact on an intestinal ecosystem with potential implications for human health.

## Introduction

The human large bowel is a highly complex and dynamic ecosystem harboring an immense number of microorganisms (Riedel et al., 2014) and representing one of the most metabolically active sites in our body (Slavin, 2013). A balanced microbiota composition and associated metabolic activities have beneficial effects on host health and wellbeing (Topping and Clifton, 2001; Clemente et al., 2012). Although a remarkable variability in the composition of the intestinal microbiota across individuals is observed (Turnbaugh et al., 2010), the major phyla and the functionality encoded by this community are quite redundant (Turnbaugh et al., 2009; Human Microbiome Project, 2012). An essential function is the metabolic capacity to ferment complex dietary carbohydrates such resistant starch (RS) (Guarner and Malagelada, 2003). RS is defined as the fraction of food-derived starch that is resistant to digestion by host amylases in the upper digestive tract and transits intact to the large bowel, where it serves as a substrate for microbial growth and is transformed to short-chain fatty acids (SCFA; butyrate, propionate and acetate; Asp, 1992; Englyst et al., 1996). Besides several other functions, SCFAs are known to play a crucial role in host physiology (Koh et al., 2016; Morrison and Preston, 2016).

Several pathologies including chronic inflammatory diseases, allergies and colorectal cancer were shown to be associated with dysbiosis of the intestinal microbiota (Sekirov et al., 2010). On the other hand, administration of prebiotics such as RS may alleviate symptoms of inflammatory bowel disease (Jacobasch et al., 1999; Bassaganya-Riera et al., 2011) and reduce the risk for colorectal cancer (Hylla et al., 1998). Although the mechanisms behind these observations are not fully understood, it has been proposed that an increase in abundance of specific bacterial groups and their fermentation products following RS administration might mediate these effects (Zhang and Davies, 2016). For these reasons, application of RS may have clinical relevance in the treatment or prevention of these diseases (Holmes et al., 2012; Higgins and Brown, 2013). However, our understanding of the fate of ingested RS and the microbial populations utilizing RS in large intestine of humans and other (model) systems is rather limited.

Early cultivation-based studies described human gut bacteria able to grow on starch within the *Bacteroidetes, Firmicutes*, and *Actinobacteria* phyla, with a particular high number of isolates assigned to *Bacteroides* spp. (Salyers et al., 1977a,b). These results were supported by studies in different animal models showing increased levels of *Bifidobacterium, Ruminococcus, Bacteroides, Lactobacillus, Eubacterium, Akkermansia, Allobaculum, Roseburia*, and *Prevotella* upon administration of RS (Kleessen et al., 1997; Jacobasch et al., 1999; Silvi et al., 1999; Le Blay et al., 2003; Young et al., 2012; Tachon et al., 2013; Umu et al., 2015). Various of these organisms were shown to encode genes for starch utilization and may therefore be able to directly breakdown and utilize RS (Xu et al., 2003, 2007; Martens et al., 2009; Ze et al., 2015). Additionally, other groups of bacteria may show an increased abundance following administration of RS by cross-feeding on mono- and oligomers derived from RS-degradation by other bacteria or their metabolic end-products as shown *in vitro* (Belenguer et al., 2006; Ze et al., 2012; Rios-Covian et al., 2015). Collectively, these studies demonstrate the potential of RS to modify the composition of the intestinal microbiota.

With the advent of next-generation sequencing approaches targeting 16S rRNA gene sequences, profound insights into the diversity and dynamics of the colonic microbiota have been gained (Eckburg et al., 2005; Qin et al., 2010). However, approaches to profile the microbial composition solely based on sequencing of 16S rRNA genes lack the ability to directly link the microbiota to metabolic capacities, such as the assimilation of specific dietary fibers or (prebiotic) carbohydrates. A comprehensive analysis of the dynamics of prebiotic carbohydrate metabolism and of the mechanisms behind their impact on health and disease requires techniques that allow the identification of metabolically active groups of microorganisms within complex (intestinal) microbiotas.

Nucleic-acid based stable isotope probing (SIP) has become a valuable tool in environmental microbial ecology (Radajewski et al., 2000). This technique enables researchers to unravel the phylogeny of microorganisms that metabolize a specific, isotope-labeled (e.g., ^13^C) compound under *in situ* conditions (Boschker et al., 1998; Dumont and Murrell, 2005; Egert et al., 2006; Neufeld et al., 2007). Metabolically active microorganisms incorporate the stable isotope into biomass, including DNA and RNA. Selective recovery of labeled nucleic acids that allow phylogenetic analysis then facilitates identification of substrate-utilizing microorganisms. In particular, the use of RNA-SIP (Manefield et al., 2002b) provides advantages, as RNA is an indicator for cellular activity and, unlike DNA, is independent of cell division. Moreover, due to a greater synthesis rate of RNA, measurable amounts of ^13^C-labeled RNA in metabolically active organisms can be obtained in shorter time frames than DNA (Manefield et al., 2002a; Dumont and Murrell, 2005).

More recently, this methodology has also been introduced into the field of gut microbial ecology and was shown to be appropriate to study assimilation processes of simple and complex carbohydrates *in situ* (Egert et al., 2007; Kovatcheva-Datchary et al., 2009; Tannock et al., 2014; Young et al., 2015; Herrmann et al., 2017b). The purpose of this study was to establish RNA-SIP for identification of bacterial populations in murine feces involved in the assimilation of stable [U^13^C]-labeled potato starch, a source of RS, and to track the ^13^C label in the products of fermentation in an *in vitro* system using fecal samples of mice origin. We observed an incorporation of ^13^C into 16S rRNA after 2 h of incubation. Comparing ^13^C-labeled and unlabeled 16S rRNA pools revealed significant differences in their community structure. In addition, by monitoring specific fermentation products in culture supernatants using high performance liquid chromatography coupled to isotope ratio mass spectrometry (HPLC-IRMS), incorporation of the ^13^C-label into fermentation metabolites was observed, albeit at low amounts. Our study provides a basis for subsequent *in vivo* investigations, particularly in murine models, to address RS fermentation processes directly in the intestinal environment.

## Materials and methods

### Collection and cultivation of murine fecal samples

Essentially, experiments were carried out as described previously (Herrmann et al., 2017b). In brief, fresh fecal pellets from eight healthy C57BL/6J mice were collected within 5 h of defecation. Since the aim of our study was to establish RNA-SIP for identification of murine fecal bacteria able to utilize RS instead of determining inter-individual variation in the populations of these bacteria, we opted for a pooling strategy. Animals were bred and housed at the animal facility of the University of Ulm in a specific pathogen-free (SPF) environment and given a standard laboratory diet with water *ad libitum*. Mice were routinely bred to maintain the colony of C57BL76 mice at the animal facility of the university and are not used for animal experimentation unless under a specific ethical approval for other studies. Collection of fecal samples from the cages does not constitute an invasive treatment, physical distress or even pain to animals and is thus not subject to ethics approval. Collected fecal pellets were pooled (total weight 1.9 g) and mixed with 12.7 mL of sterile, deoxygenated, and pre-warmed (37°C) M9 minimal medium (Smith and Levine, 1964) without glucose supplemented with 2 mg L^−1^ thiamin and 1 g L^−1^ casamino acids resulting in a 15% (w/v) fecal slurry (0 h, control). Deoxygenation of the medium prior to experiments was achieved by incubation in airtight jars under anaerobic conditions generated using AnaeroGen sachets (Merck, Darmstadt, Germany) 24 h prior to experiments. For each incubation, 1 mL of slurry material was diluted 1:2 with M9 minimal medium containing either 14.4 mg [^12^C] or 15 mg of uniformly (98%) labeled [U^13^C] potato starch, yielding a final concentration of 40 mM glucose unit equivalents and 7.5% (w/v) of fecal material. Both [^12^C] and [U^13^C]starch were obtained from the same manufacturer (IsoLife, Wageningen, Netherlands), were isolated from the same potato variety, and therefore presumably contained similar levels of RS. Diluted fecal material was incubated with the native [^12^C] or [U^13^C]potato starch in single 15-mL reaction tubes at 37°C for 2 h or 4 h in airtight jars under anaerobic conditions generated using AnaeroGen sachets (Merck, Darmstadt, Germany). AnaeroGen sachet will reduce the oxygen level in the jar to below 1% within 30 min (information by the supplier). Incubation times of 2 and 4 h were chosen on the basis of recent experiments from a previously published RNA-SIP study of our group with murine fecal slurries and labeled glucose as substrate (Herrmann et al., 2017b). The results of these preliminary experiments showed that even with an easily accessible substrate such as glucose, sampling times shorter than 2 h after addition of the substrate yielded only negligible amounts of labeled RNA. This indicates that the earliest time point for detection of bacteria that have incorporated the isotope label into RNA (and thus represent glucose utilizing bacteria) is 2 h. Based on these observations, 2 and 4 h were considered reasonable time points to identify RS-utilizing bacteria. Incubations for each condition (0 h, 2 h-^12^C, 2 h-^13^C, 4 h-^12^C, 4 h-^13^C) were carried out in technical duplicates using the same fecal slurry.

### Nucleic acid extraction, isopycnic density gradient ultracentrifugation, gradient fractionation

Total RNA from each sample was extracted and residual genomic DNA was removed as described before (Herrmann et al., 2017b). After elution and quantification, absence of DNA was verified by PCR and total RNA was subsequently loaded into a cesium trifluoroacetate (CsTFA) centrifugation solution for density-dependent resolution by gradient ultracentrifugation following a previously published protocol (Herrmann et al., 2017a,b). Afterwards, gradients were fractionated and the buoyant density (BD) of each fraction was determined as previously described (Herrmann et al., 2017b). Briefly, CsTFA (average BD = 1.793 g mL^−1^) was loaded with ~700 ng RNA, filled into 8 mL Quick-Seal polypropylene tubes (BeckmanCoulter Inc., Krefeld, Germany) which were subsequently sealed. Sealed tubes were placed in an MLN-80 rotor in an Optima MAX-XP bench-top ultracentrifuge (both BeckmanCoulter Inc.) and subjected to ultracentrifugation at 123,100 × g (45,000 rpm) and 20°C for 67 h to establish density gradients. After centrifugation, density gradients were fractionated into 16 equal fractions (each 0.5 mL) by displacement with water from the top of the tube under a consistent flow rate of 1 mL min^−1^ using a syringe pump (World Precision Instruments, Berlin, Germany). BD of each fraction was determined by measuring the refraction of the solution using a refractometer (Reichert, Depew, NY, USA) and correlation to a previously established calibration curve. For subsequent analysis, RNA from each fraction was precipitated, washed and re-dissolved in nuclease-free water.

### Reverse transcription, quantification of 16S rRNA in gradient fractions, 16S rRNA amplicon library construction and sequencing

The amount of bacterial 16S rRNA in each gradient fraction was determined by quantitative reverse transcription polymerase chain reaction (RT-qPCR) (Lueders et al., 2004) in a two-step assay. Total RNA in 10 μL of each gradient fraction, was reverse transcribed to complementary DNA (cDNA) using the SuperScript VILO cDNA Synthesis Kit (Life Technologies, Darmstadt, Germany) according to the manufacturer's protocol. The cDNA was then used as template in qPCR reactions with universal bacterial primers F_Bact 1369 (5′-CGG TGA ATA CGT TCC CGG-3′) and R_Prok1492 (5′-TAC GGC TAC CTT GTT ACG ACT T-3′) (Furet et al., 2009) on a Light-Cycler® 480 system (Roche, Mannheim, Germany). A PCR reaction of 25 μL contained 12.5 μL of 2x Maxima SYBR Green/ROX qPCR Master Mix (Life Technologies), 0.15 μL of each primer (50 μM; Metabion, Planegg/Steinkirchen, Germany), 0.25 μL bovine serum albumin (BSA; 20 mg mL^−1^; Roche), 9.95 μL of nuclease-free water and 2 μL of cDNA template. Amplification was carried out with the following thermal profile: 95°C for 10 min followed by 40 cycles of 95°C for 15 s (denaturation), 60°C for 30 s (annealing), and 72°C for 30 s (elongation). Serial dilutions of purified 16S rRNA gene amplicons of *Escherichia coli* K12 were used as an internal standard. Nucleic acid concentrations were calculated against the standard curve using the Light-Cycler® 480 software (version 1.5). To allow comparison between different gradients and samples, the 16S rRNA content in each fraction was normalized according to a procedure used by us (Herrmann et al., 2017b) and others (Lueders et al., 2004; Hatamoto et al., 2007). RNA content in different fractions is expressed as proportion (%) of the RNA content in the fraction of the same sample with the highest RNA concentration, which was set to 100%.

**Figure 1:**
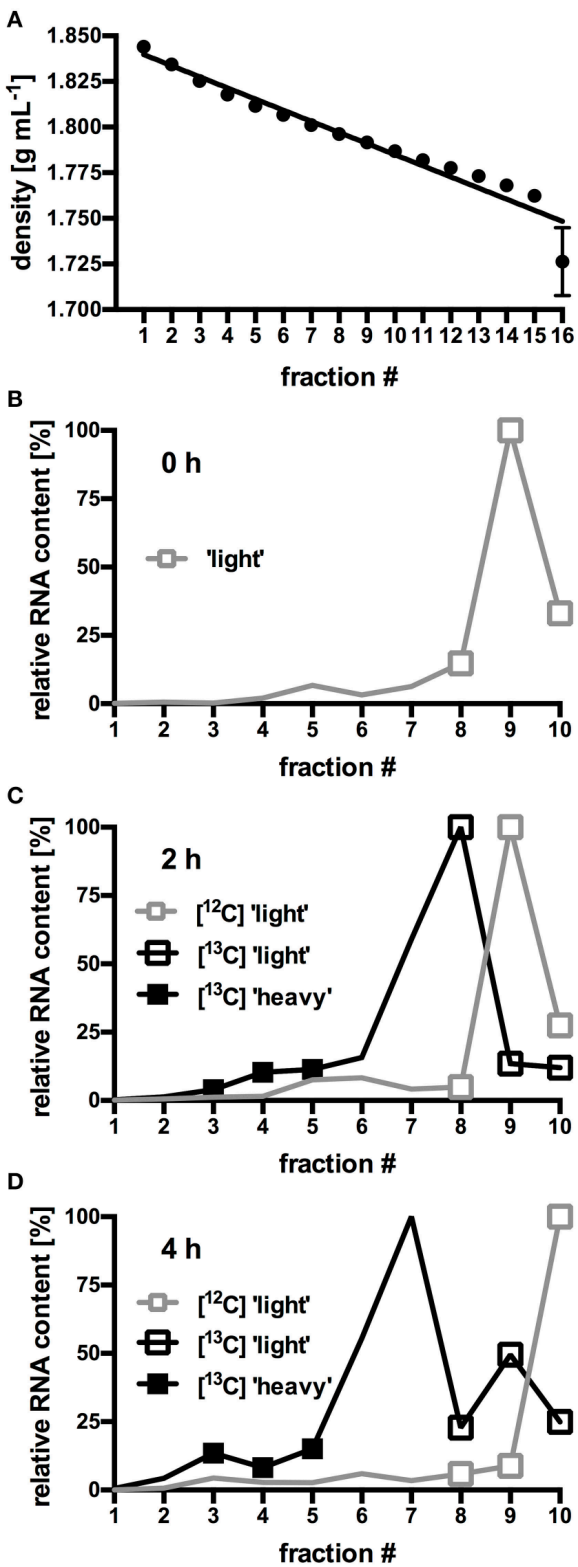
Buoyant density (BD) curves from isopycnic density gradients **(A)**. Values represent mean and standard error (SEM) of 10 gradients. For each fraction, SEM was ≤0.02 g mL^−1^, error bars for fraction 1–15 are smaller than the symbols. **(B–D)** Relative amount of RNA in selected gradient fractions. RNA was isolated from fresh fecal slurry (0 h; **B**), and from slurries incubated with either [^12^C]starch or [^13^C]starch for 2 h **(C)** or 4 h **(D)**. Values represent mean of duplicate incubations per gradient fraction. To facilitate comparison between different gradients, RNA content in each fraction was expressed as proportion (%) of the total amount of RNA found in the fraction containing the highest RNA concentration (Herrmann et al., 2017b). “Heavy” and “light” RNA from the fractions highlighted by symbols were selected for further analysis of microbiota composition by NGS.

In order to identify the bacterial populations involved in RS assimilation, 16S rRNA gene amplicon libraries were constructed from the density-resolved and reverse-transcribed rRNA. Figures 1B–D shows the density fractions which were selected for sequencing. Gradient fractions 8–10 (Figures 1B–D) contained RNA in all preparations. Thus, they presumably represented unlabeled RNA and were designated “light” SIP fractions. A discernable amount of RNA in gradient fractions 3–5 (Figures 1B–D) were only obtained from the [U^13^C]starch incubations. These fractions presumably contained heavy, [^13^C]-labeled RNA of bacteria that actively metabolized labeled [U^13^C]starch and were designated “heavy” SIP fraction. Sequencing libraries of the respective density fractions were constructed from all incubations.

Amplicon libraries representing the V3 and V4 regions of the bacterial 16S rRNA gene were prepared as previously described (Herrmann et al., 2017b). Briefly, 16S rRNA genes were amplified from cDNA of gradient fractions in a first PCR step using the locus-specific primer set S-D-Bact-0341-b-S-17 and S-D-Bact-0785-a-A-21 (Klindworth et al., 2013), to which overhang adaptor sequence tails were added. After purification of the obtained PCR products a second PCR step was performed to anneal unique dual-index barcodes with Illumina sequencing adaptors (Nextera XT index kit; Illumina, Eindhoven, Netherlands) to the amplicon target. The obtained 16S amplicon libraries were subjected to bead-purification using Agencourt AMPure XP beads (BeckmanCoulter Inc.) and size of the amplicons (~630 bp) was verified with a Bioanalyzer DNA 1000 chip (Agilent Technologies GmbH, Waldbronn, Germany). Quantities of each library were fluorometrically assessed using the Qubit dsDNA HS assay kit (Life Technologies). Finally, the uniquely indexed 16S amplicon libraries were pooled in equimolar amounts (4 nM) and sequenced in technical duplicates on an Illumina MiSeq platform (Illumina, Eindhoven, Netherlands) by Eurofins Genomic (Eurofins Genomic, Ebersberg, Germany) using the Illumina MiSeq 600 cycle Kit_V3 chemistry (2 x 300 bp PE; Illumina).

### Sequencing analysis and statistics

Sequencing data were processed with QIIME 1.8 (Caporaso et al., 2010) as previously described (Herrmann et al., 2017b). To measure the bacterial diversity in the different density fractions, diversity analyses were performed using the core_diversity_analyses.py script. Faith's phylogenetic diversity estimate was calculated using the minimum number of reads (6397) across 10 iterations.

Statistical analyses of the sequencing data were performed using R 3.3.2 (R Core Team, 2015) to analyze the community structure represented by the cDNA of the 16S rRNA species present in the “heavy” and “light” fractions per time point and treatment; all presented results on the taxonomic composition were first averaged across the sequencing duplicates to obtain the community per fraction. Respective fractions were then averaged across both incubation duplicates (except for fraction 8, which is represented by only one incubation) and finally across the designated different density fractions (“heavy” and “light”), resulting in a total of seven different observation groups, each represented by three fractions (0 h “light,” 2 h [^12^C] “light,” 4 h [^12^C] “light,” 2 h [^13^C] “light,” 2 h [^13^C] “heavy,” 4 h [^13^C] “light,” and 4 h [^13^C] “heavy”).

Differences in the prevalence of bacterial taxa in the “light” SIP fractions during the course of the ^12^C-control incubations were assessed using non-parametric permutation ANOVA with time as factor. Differences in the prevalence of bacterial taxa between “heavy” and “light” SIP fractions of the [U^13^C]starch treatments were assessed using two-factor permutation ANOVA with time and density as factors. Both tests were implemented using the perm.anova function in the RVAideMemoire package for R (Hervé, 2015) with 1000 permutations. Adjustment of *P*-values for multiple testing was performed using the Benjamini & Hochberg false discovery rate (FDR) method, with FDR < 0.05 considered significant. Differences in alpha diversity were analyzed using two-factor ANOVA with incubation time and density as factors. Differences with a *P*-value < 0.05 were considered significant. Hierarchical cluster analysis of bacterial profiles was performed using distances calculated from centered Pearson's correlation and average linkage clustering.

All sequencing data were submitted to GenBank and are publicly available under the accession number PRJNA376059.

### Profiling of specific fermentation products during starch fermentation

Total concentration and ^13^C-enrichment of lactate, acetate, propionate, butyrate and isobutyrate stemming from bacterial fermentation in the fecal slurries were monitored using HPLC-C-IRMS (Thermoquest, Bremen, Germany) as described before (Conrad et al., 2007; Liu and Conrad, 2011).

Concentrations and retention times of acetate, propionate, butyrate and isobutyrate were determined by comparison with unlabeled standards. The isotopic signal of the ^13^C/^12^C ratio detected in the IRMS was calibrated with a CO_2_ gas standard, which was referenced against a methyl stearate working standard and calibrated at the Max Planck Institute for Biogeochemistry, Jena, Germany (courtesy W.A. Brand). The proportion of labeled SCFA was calculated as atom percent excess (APE). This was achieved by measuring concentration of ^13^C-labeled SCFA in untreated and [^12^C]starch-treated samples. Consistent with the natural abundance of ^13^C, a stable proportion of 1.08% of all SCFA in these samples was ^13^C-labeled. This value was subtracted from the percentage of labeled SCFA in samples of [U^13^C]starch incubations to obtain APE.

## Results

### RNA recovery and PCR amplification of 16S rRNA

In order to identify bacteria able to utilize RS, slurries prepared from fresh murine fecal pellets were incubated with [U^13^C]potato starch and incorporation of the ^13^C-label into bacterial RNA was determined. The fractionated centrifugation gradients showed decreasing average BDs (Figure 1A) ranging from 1.844 g mL^−1^ (fraction 1) to 1.726 g mL^−1^ (fraction 16) which indicated an adequately linear gradient formation.

In gradients containing only unlabeled [^12^C]RNA species originating from fresh fecal slurry (0 h; Figure 1B) and from slurries incubated with [^12^C]starch for 2 h (Figure 1C) and 4 h (Figure 1D), an almost identical RNA distribution pattern was observed: The bulk of the unlabeled RNA species was present in low density fractions (≤1.796 g mL^−1^; ≥fraction 8) with peak amounts detected in fraction 9 (1.792 g mL^−1^) after 0 h and 2 h of incubation and in fraction 10 (1.787 g mL^−1^) after 4 h of incubation. In gradients containing RNA extracted from fecal slurries incubated with the [U^13^C]starch, RNA content slightly shifted toward higher density fractions (≥1.796 g mL^−1^; ≤fraction 8) with the highest concentration accumulated in fraction 8 and 7 (1.796–1.801 g mL^−1^) after 2 and 4 h of incubation respectively (Figures 1C and D). Moreover, in these samples a second, smaller peak in RNA quantity was observed in fraction 3–5 (1.812–1.825 g mL^−1^).

### Bacterial structure of fresh and incubated fecal communities

Sequencing of the selected RNA-SIP fractions generated a total of 2,407,933 paired end sequences with an average of 32,985 sequences per sample (minimum 6,397, maximum 109,654, standard deviation 20,421). The obtained 16S rRNA gene sequences in the entire dataset were affiliated with nine phyla, 16 classes, 25 orders, 52 families and 98 genera.

**Figure 2:**
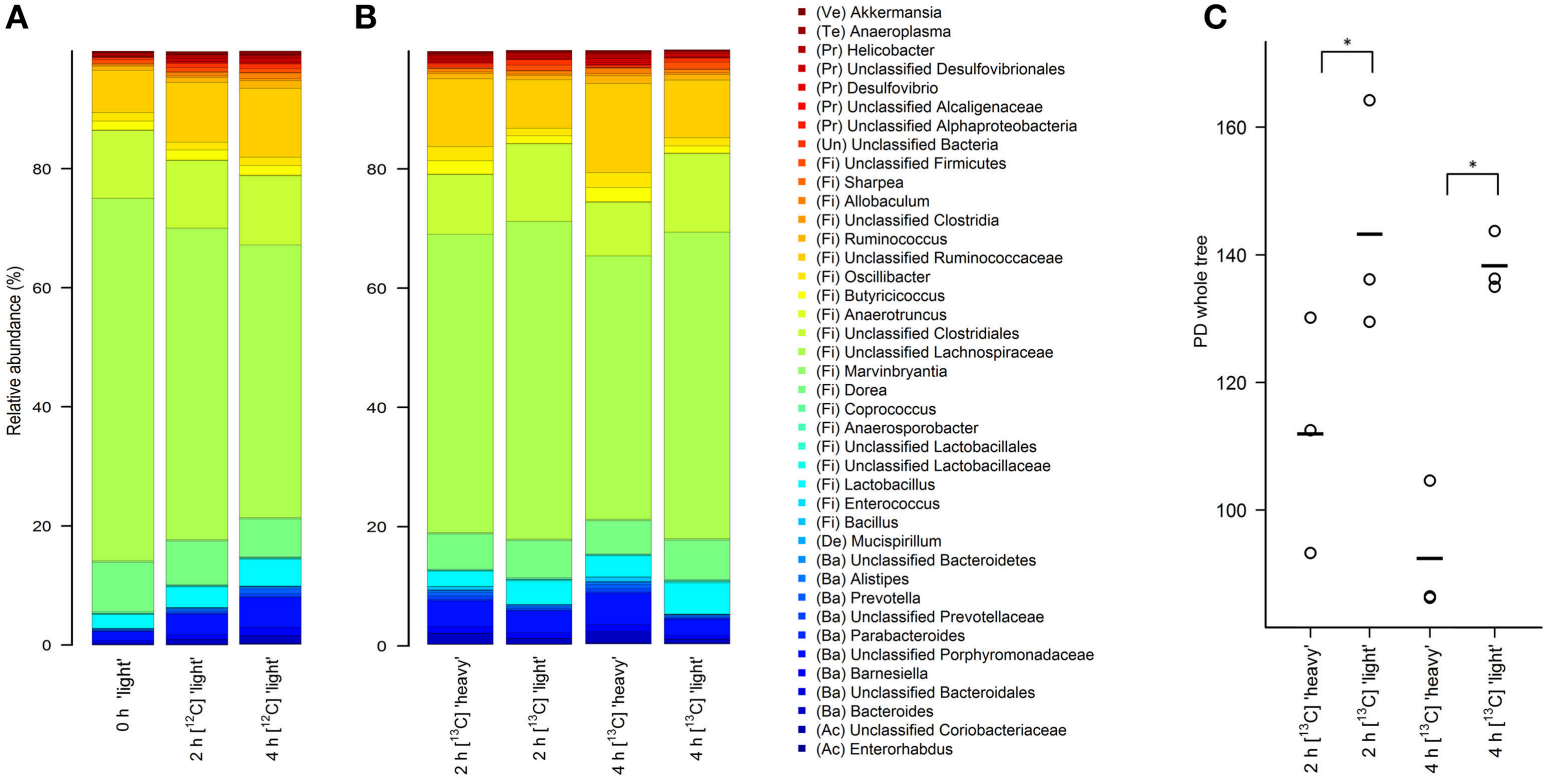
Stacked barplots showing the average bacterial microbiota composition represented by 16S rRNA gene amplicons in different density SIP fractions. RNA was isolated from fresh fecal content (0 h) or after 2 and 4 h of incubation in the presence of unlabeled [^12^C]starch **(A)** or isotope-labeled [U^13^C]starch **(B)**. The 40 taxa with highest mean relative abundance across all samples are shown. **(C)** Faith's phylogenetic diversity estimate in “heavy” and “light” RNA fractions of the [U^13^C]starch fed communities after 2 and 4 h of incubation. ^*^Indicates significance in complexity at *p* < 0.01 as calculated by two-factor ANOVA with time and density as factors.

“Light” fractions collected from the unlabeled [^12^C]starch fermentations were analyzed to show how the overall fecal community changed during the course of incubation. In line with a previous study using fecal slurries of animals housed in the same facility (Herrmann et al., 2017b), Firmicutes was the dominant phylum in fresh fecal content followed by Bacteroidetes and Proteobacteria (Table 1, 0 h). Verrucomicrobia, Actinobacteria, Tenericutes and Deferribacteres were detected at low frequencies, and 0.4% of the sequences remained unclassified. Clostridia was the most prevalent class with sequences affiliated to unclassified Lachnospiraceae, unclassified Clostridiales, Dorea and unclassified Ruminococcaceae dominating the community (Figure 2A, 0 h “light”). In almost equal relative frequencies, Bacilli and Bacteroidia were the second most dominant classes with Lactobacillus and unclassified Porphyromonadaceae as their most abundant representatives (Table 1 and Figure 2A). Structural changes in the bacterial community were observed over the course of incubation in the ^12^C-control fermentations (Table 1 and Figure 2A). A significant drop in Firmicutes (FDR = 0.02), mainly caused by reduction of the abundant unclassified Lachnospiraceae, was observed. This drop was accompanied by an increase in the relative abundances of the majority of the other taxa detected. The most prominent increase in abundance was observed for Bacteroidetes (FDR = 0.02) and Proteobacteria (FDR = 0.064). However, community profiles were still dominated by Firmicutes.

**Table 1:** Relative abundances of bacterial taxa represented by 16S rRNA gene amplicons in “light” RNA-SIP fractions obtained from fresh fecal slurry material (0 h) or after incubation with [^12^C]starch for 2 and 4 h.

	**Fresh fecal material and [**^**12**^**C]starch control incubations**							
	**Relative abundances (%)**							
	**0 h**		**2 h**		**4 h**			
							**Time**	
**Bacterial taxon**	**Mean**	**SEM**	**Mean**	**SEM**	**Mean**	**SEM**	***P*-value**	**FDR**
Firmicutes	95.68	0.45	90.89	0.73	87.18	0.58	0.003	0.020
*Clostridia*	92.30	1.27	85.79	2.05	80.61	0.90	0.011	0.027
*Bacilli*	2.51	0.61	3.69	1.10	4.80	0.44	0.205	0.236
Bacteroidetes	2.66	0.32	6.11	0.51	9.49	0.50	0.005	0.020
*Bacteroida*	2.62	0.32	6.04	0.51	9.40	0.50	0.001	0.015
Proteobacteria	0.78	0.10	1.54	0.16	1.53	0.15	0.036	0.064
Uc. Bacteria	0.40	0.08	0.72	0.18	0.74	0.25	0.432	0.432
Verrucomicrobia	0.29	0.04	0.43	0.06	0.58	0.06	0.034	0.064
Actinobacteria	0.10	0.02	0.16	0.02	0.26	0.05	0.056	0.064
Tenericutes	0.07	0.01	0.11	0.02	0.17	0.02	0.043	0.064
Deferribacteres	0.02	0	0.04	0	0.05	0.01	0.049	0.064
TM7	n. d	0	n. d	0	0.01	0	–	–

*Values represent mean (n = 3 fractions) and standard error of the mean (SEM). P-value indicates permutation ANOVA significance with time as factor. False Discovery Rate (FDR) indicates multiple testing adjusted P-value. Uc., Unclassified; n.d., not detected*.

### Prolific users of resistant starch derived carbon

**Figure 3:**
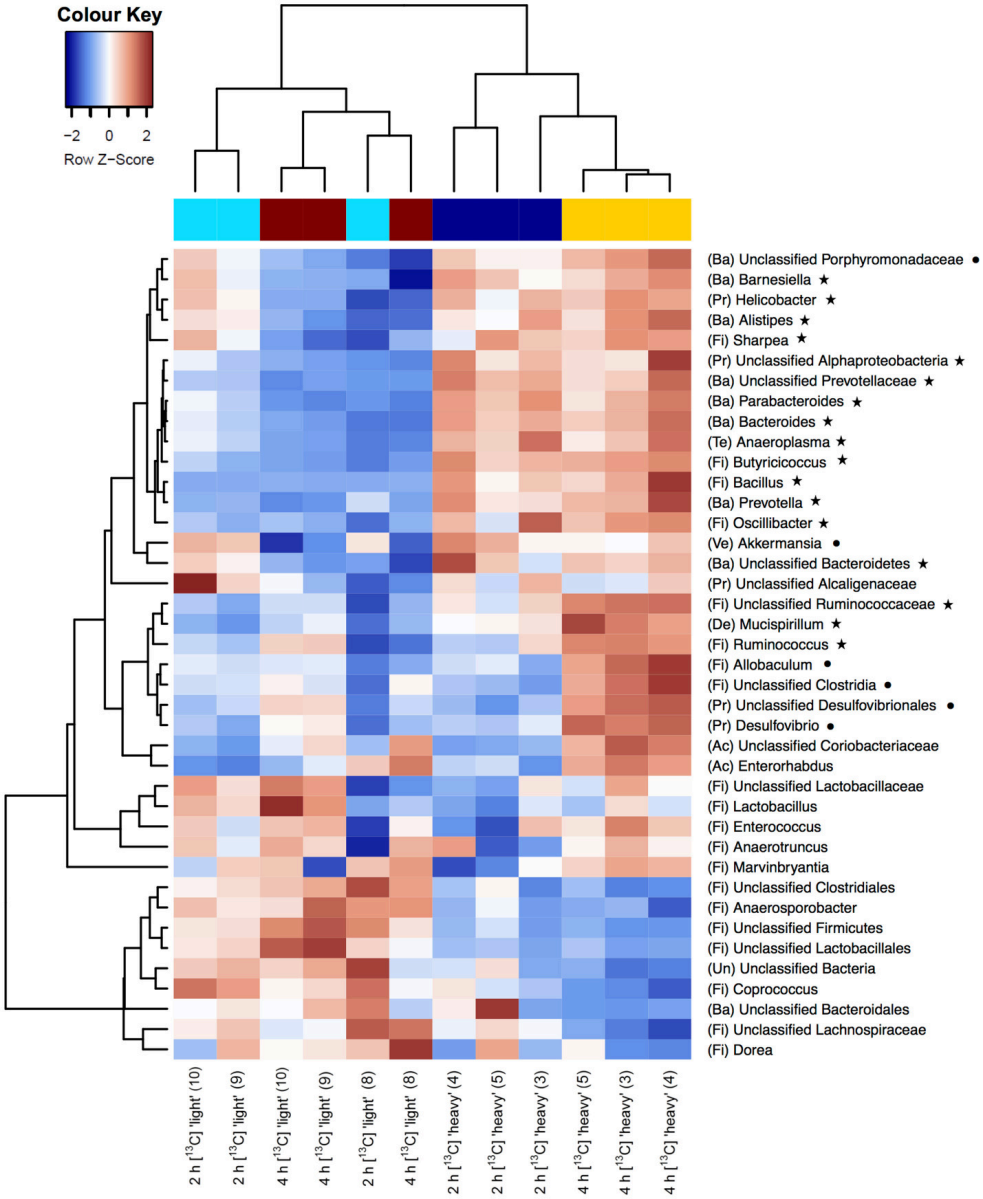
Heatmaps showing hierarchical clustering of bacterial community composition profiles of the 40 most abundant taxa represented by 16S rRNA gene amplicons per analyzed sample of the “heavy” and “light” SIP fractions. RNA was isolated from fecal slurries following incubation with [U^13^C]starch after 2 h or 4 h. Heatmap color (blue to dark red) displays the row scaled relative abundance of each taxon across all samples. The number in parentheses indicates the corresponding fraction number. Letters in parentheses preceding taxonomic labels indicate the phylum (Ac, Actinobacteria; Ba, Bacteroidetes; De, Deferribacteres; Fi, Firmicutes; Pr, Proteobacteria; Ve, Verrucomicrobia; Un, Unclassified). Symbols behind taxonomic labels indicate significant higher relative abundance at FDR ≤ 0.05 of taxa in “heavy” fractions compared with “light” fractions after 2 h (⋆) or 4 h (•).

As a first step toward the identification of bacterial populations involved in RS fermentation, community profiles in “heavy” and “light” density fractions of the ^13^C-fed cultures were analyzed for proportional differences of individual taxa (Wüst et al., 2011; Young et al., 2015). Previous studies have shown that the amount of ^13^C in RNA is directly linked to the bouyant density and RNA molecules with different proportions of ^13^C can be separated by density centrifugation and fractionation (Manefield et al., 2002b). Moreover, nucleic acid samples from RNA- and DNA-SIP experiments with increased BD also had an enrichment in ^13^C-contents, i.e., higher density fractions indeed contained ^13^C-labeled RNA/DNA, as measured independently by IRMS (Manefield et al., 2002a; Shao et al., 2014). We therefore considered it reasonable to assume that “heavy” fractions from samples incubated with [U^13^C]starch contained isotope labeled RNA. Sequencing profiles revealed a complex bacterial community structure consisting of many taxa in the “heavy” fractions after 2 and 4 h of incubation in the presence of [U^13^C]starch (Figure 2B). However, in these fractions alpha diversity was significantly (*P* < 0.01) lower compared to the corresponding “light” fractions at both time points (Figure 2C). Moreover, hierarchical clustering analysis showed bacterial profiles from the “heavy” fractions were clearly separated from the profile of the “light” fractions as well as a delineation between the “heavy” fractions of both time points (Figure 3).

At the phylum level, the relative abundance of Bacteroidetes was considerably increased in “heavy” SIP fractions (FDR = 0.003) compared with their “light” counterpart. Similarly, proportions of Proteobacteria were also enriched in “heavy” gradient fractions (FDR = 0.017), whereas a significant decrease in Firmicutes was detected (FDR = 0.005) (Table 2 and Supplementary Figure S1).

**Table 2:** Prevalence of bacterial taxa represented by 16S rRNA gene amplicons in “light” and “heavy” RNA-SIP fractions obtained from incubations with [^13^C]starch for 2 and 4 h.

	**[**^**13**^**C]starch incubations**												
	**Relative abundances (%)**												
	**2 h**				**4 h**								
	**Heavy**		**Light**		**Heavy**		**Light**		**Density**		**Time**		**Density × Time**
**Bacterial taxon**	**Mean**	**SEM**	**Mean**	**SEM**	**Mean**	**SEM**	**Mean**	**SEM**	***P*-value**	**FDR**	***P*-value**	**FDR**	***P*-value**
Firmicutes	88.03	0.50	90.68	1.20	86.43	0.85	92.82	0.77	0.002	0.005	0.789	0.789	0.072
Uc. *Lachnospiraceae*	50.10	0.72	53.30	1.97	44.18	0.96	51.44	2.29	0.013	0.021	0.055	0.169	0.249
Uc. *Ruminococcaceae*	11.40	0.65	8.14	0.92	14.91	0.18	9.68	0.38	0.001	0.005	0.007	0.035	0.131
Uc. *Clostridiales*	9.99	0.83	13.02	1.20	8.99	0.41	13.15	0.23	0.001	0.005	0.588	0.790	0.529
*Oscillibacter*	2.33	0.36	1.26	0.16	2.49	0.12	1.37	0.04	0.003	0.007	0.508	0.753	0.892
*Butyricicoccus*	2.27	0.14	1.27	0.15	2.39	0.07	1.19	0.01	0.002	0.006	0.770	0.894	0.397
*Ruminococcus*	0.90	0.10	0.68	0.12	1.34	0.02	0.93	0.19	0.037	0.051	0.030	0.109	0.443
*Allobaculum*	0.57	0.03	0.55	0.05	0.88	0.05	0.57	0.04	0.006	0.013	0.012	0.053	0.022
*Bacillus*	0.53	0.10	0	0	0.72	0.18	0	0	0.002	0.006	0.365	0.583	0.365
Uc. *Clostridia*	0.25	0.01	0.25	0.02	0.38	0.02	0.29	0.01	0.035	0.050	0.002	0.020	0.020
Uc. *Firmicutes*	0.24	0.11	0.94	0.16	0.08	0.05	1.19	0.22	0.001	0.005	0.782	0.894	0.197
Bacteroidetes	9.04	0.39	6.58	1.00	10.24	0.71	4.80	0.57	0.001	0.003	0.660	0.734	0.066
Uc. *Porphyromonadaceae*	4.31	0.17	3.69	0.61	5.27	0.27	2.75	0.39	0.007	0.014	0.980	0.980	0.044
*Bacteroides*	1.76	0.09	0.87	0.21	1.85	0.18	0.61	0.07	0.002	0.006	0.552	0.789	0.278
*Barnesiella*	1.08	0.07	0.91	0.12	1.14	0.06	0.63	0.11	0.008	0.015	0.265	0.529	0.100
*Prevotella*	0.61	0.06	0.37	0.03	0.72	0.07	0.28	0.02	0.001	0.005	0.835	0.903	0.082
Uc. *Prevotellaceae*	0.60	0.04	0.27	0.03	0.56	0.07	0.21	0.01	0.001	0.005	0.309	0.552	0.701
Proteobacteria	1.57	0.15	1.25	0.27	2.14	0.12	1.23	0.13	0.014	0.017	0.154	0.256	0.130
*Helicobacter*	0.71	0.06	0.55	0.14	0.79	0.03	0.41	0.04	0.016	0.025	0.631	0.809	0.197
Uc. *Desulfovibrionales*	0.34	0.03	0.34	0.05	0.72	0.03	0.50	0.05	0.040	0.053	0.001	0.013	0.038
Other													
Uc. *Bacteria*	0.50	0.10	0.88	0.12	0.26	0.05	0.67	0.10	0.006	0.013	0.044	0.147	0.863

*Values represent mean (n = 3 fractions) and standard error of the mean (SEM). Presented are taxa that show significant differences in mean relative abundances between the “heavy” and “light” gradient fractions of the ^13^C-labeled community among the 20 most abundant taxa detected. P-value indicates two-factor permutation ANOVA significance with density and time as factors. False Discovery Rate (FDR) indicates multiple testing adjusted P-value. Uc, unclassified; n.d., not detected*.

Among the 40 most abundant taxa, identified at the lowest taxonomic level available, 23 showed a significantly increased prevalence in the “heavy” SIP fractions (FDR < 0.05) compared to the “light” fractions. After 2 h of incubation, the taxa that dominated the community were assigned to unclassified Ruminococcaceae, unclassified Prevotellaceae and Prevotella spp. (FDR = 0.005; Table 2 and Supplementary Figure S1). In addition, a diverse array of sequences affiliated with Butyricicoccus, Bacteroides, Bacillus (all FDR = 0.006), Oscillibacter (FDR = 0.007), Barnesiella (FDR = 0.015), Helicobacter (FDR = 0.025) and, to a lesser extent, *Ruminococcus* spp. (FDR = 0.051) showed increased relative abundances in the “heavy” fractions (Table 2). Moreover, unclassified Ruminococcaceae (FDR = 0.035) and, to a lesser extent, *Ruminococcus* (FDR = 0.109) increased in relative proportions between 2 and 4 h of incubation (Table 2).

Based on a significant interaction *P*-value obtained between density and time (all *P* ≤ 0.044), Allobaculum (FDR = 0.013), unclassified Porphyromonadaceae (FDR = 0.014), unclassified Clostridia (FDR = 0.05) and, to a lesser extent, unclassified Desulfovibrionales (FDR = 0.053) were detected in increased proportions in the “heavy” fractions after 4 h of incubation in the presence of the [U^13^C]starch (Table 2). The increase of these taxa was associated with a decrease in the relative abundance of unclassified Clostridiales, unclassified Firmicutes (both FDR = 0.005), unclassified Bacteria (FDR = 0.013), unclassified Lachnospiraceae (FDR = 0.021) and some minor abundant taxa (Table 2 and Supplementary Figure S1).

### ^13^C-labeled metabolite production

HPLC-IRMS analysis was used to trace the ^13^C-label derived from the [U^13^C]starch into fermentation metabolites produced by the murine fecal microbiota from the same incubations as used for the RNA isolation. An almost identical pattern was observed in fermentations with [U^13^C]starch and [^12^C]starch with acetate (~12 mM), propionate (~4 mM), and butyrate (~2 mM) being the most abundant SCFA. The branched-chain SCFA (BCFA) iso-butyrate was also detected, albeit in lower amounts (Table 3). Interestingly, lactate (0.61 mM) was only detected in fresh fecal slurry at the start of the fermentation process. However, during the course of fermentation, no considerable changes in concentrations of SCFA could be observed (Table 3). Nevertheless, incorporation of the isotope-label from the [U^13^C]starch into the SCFA, expressed as atom percent excess (APE), could be detected consistently (Table 3). After 2 h, 0.95, 1.46, and 0.39% of labeled acetate, propionate and butyrate, respectively, was derived from RS. These proportions increased to, 2.00, 2.75, and 1.11%, respectively, after 4 h of incubation.

**Table 3:** Time profiles of concentration and relative ^13^C-enrichment of metabolites in fresh fecal slurry (0 h, t_0_) and 2 (t_2_) and 4 h (t_4_) after addition of either [^12^C]starch or [U^13^C]starch at a concentration equivalent to 40 mM of glucose.

	**t_0_**	**t**_**2**_			**t**_**4**_		
**Product**	**concentration (mM)**	**concentration (mM)**		**APE**	**concentration (mM)**		**APE**
	**Fresh feces**	**[^12^C]starch**	**[^13^C]starch**	**[^13^C]starch**	**[^12^C]starch**	**[^13^C]starch**	**[^13^C]starch**
Lactate	0.61 ± 0.18	–	–	–	–	–	–
Acetate	11.89 ± 0.11	10.34 ± 0.48	11.04 ± 0.19	0.95 ± 0.03	10.67 ± 0.54	11.69 ± 0.01	2.00 ± 0.04
Propionate	1.97 ± 0.11	2.88 ± 0.16	4.20 ± 0.08	1.46 ± 0.05	2.90 ± 0.97	3.66 ± 1.82	2.75 ± 0.03
Butyrate	0.81 ± 0.14	0.94 ± 0.01	1.69 ± 0.50	0.39 ± 0.20	1.40 ± 0.70	1.09 ± 0.03	1.11 ± 0.00
Isobutyrate	0.13 ± 0.01	0.12 ± 0.00	0.12 ± 0.00	0.66 ± 0.00	0.13 ± 0.00	–	–

*The enrichment with ^13^C-atoms in the metabolites fermented on [U^13^C]starch are presented as atom percent excess (APE). Values represent mean and standard deviation (SD) of technical duplicates. -, below detection limit*.

## Discussion

The consumption of complex carbohydrates, such as RS, is known to influence colonic function and to have an impact on host health and well-being (Nugent, 2005; Birt et al., 2013). In various animal studies, ingested RS induced changes in the composition of the intestinal microbiota. For example, increased levels of *Bifidobacterium, Bacteroides, Lactobacillus*, and *Eubacterium* as well as specific fermentation end products were observed (Kleessen et al., 1997; Jacobasch et al., 1999; Silvi et al., 1999; Bird et al., 2000; Le Blay et al., 2003; Young et al., 2012). However, in most cases a direct link between specific intestinal bacteria and utilization of a particular substrate *in situ* without the need for bulk enrichments has not been demonstrated. In the present study, we used RNA-SIP together with high throughput 16S rRNA sequencing and profiling of selected ^13^C-labeled metabolites to unravel the phylogenetic identity of bacteria implicated in intestinal fermentation of RS. Fermentation experiments were performed with a single slurry obtained by pooling and homogenizing fecal pellets of eight individual mice. While this approach may not allow to identify inter-individual variations in microbial populations in response to RS, it has been demonstrated that pooling of fecal samples for microbiota profiling does not result in a pathologically altered composition of the pooled community vs. non-pooled individual samples (Aguirre et al., 2014). Pooling fecal material can thus be considered a valid approach to identify RS-utilizing bacterial groups in *in vitro* fermentations.

Obtained density spectra of the gradients were similar to gradients from other RNA-SIP studies that separated isotope-labeled [^13^C]RNA from native molecules (Egert et al., 2007; Herrmann et al., 2017b). After 2 and 4 h of incubation with [U^13^C]starch, a second peak of higher density fractions containing RNA was detected, presumably containing RNA labeled upon assimilation of the [^13^C]starch (or its degradation products) by metabolically active members of the bacterial community. The fact that only a small fraction of the total RNA of these samples was found in higher density fractions may be explained by a majority of bacteria in the system that did not utilize the labeled substrate. In addition, the fecal inoculum itself represents a rich source of unlabeled substrates that further limit uptake, utilization and incorporation of labeled RS (Egert et al., 2006). Also, oxygen levels during the experiment may have had an impact on RS fermentation. Medium was deoxygenated prior to experiments and oxygen levels in the headspace should have been reduced to below 1% within 30 min, i.e. oxygen levels typically found in the lower intestinal tract of mice (He et al., 1999). Furthermore, residual oxygen may have been removed by facultative anaerobes such as *Escherichia coli* (Marteyn et al., 2011). However, the exact oxic conditions in the system are not known and it cannot be ruled out that the low level of labeled RNA is due to incomplete anaerobiosis and subsequent metabolic inactivity of strictly anaerobic bacteria.

Amplicon libraries constructed from labeled and unlabeled 16S rRNA revealed a bacterial community composition similar to that found in murine fecal material in previous studies (Lu et al., 2016; Rausch et al., 2016; Herrmann et al., 2017b). The lower diversity detected in the “heavy,” ^13^C-labeled fractions compared with the “light” fractions suggests that a distinct subset of the fecal microbiota was actively involved in starch assimilation and/or very rapidly obtained the label by cross-feeding on fermentation products of (primary) RS-utilizers.

Our results indicate that *Prevotella, Bacteroides* as well as members of the *Ruminococcaceae* were the most prolific starch assimilators in our system. These bacterial groups have been associated previously with degradation and utilization of RS in the gastrointestinal tract of ruminants and humans (Flint et al., 2008; Ze et al., 2012; Salonen et al., 2014). Moreover, our results are in good agreement with a previous RNA-SIP study using ^13^C-labeled potato starch as a substrate for a mixed fecal human microbiota, suggesting that the major assimilators were *Ruminococcus bromii, Prevotella* spp., and *Eubacterium rectale* (Kovatcheva-Datchary et al., 2009). The authors also observed increased levels of *Bifidobacterium* spp. in their human microbiota after addition of labeled starch. We only detected very low levels of *Bifidobacterium* spp., and they were not enriched in “heavy” RNA fraction. This might be explained by the lower abundance of bifidobacteria in the murine gastrointestinal tract compared to humans (Turroni et al., 2013; Duranti et al., 2014). Furthermore, two recent studies reported increased levels of multiple *Ruminococcaceae* phylotypes as well as *E. rectale* in obese men consuming RS (Walker et al., 2011; Salonen et al., 2014) and piglets fed RS responded with an enrichment of *Prevotella-, Ruminococcus-*, and *Lachnospiraceae*-affiliated phylotypes (Umu et al., 2015). These results support the idea that indeed members of the *Prevotellaceae* and *Ruminococcaceae* were the primary RS utilizers in our *in vitro* system. This is further corroborated by (meta)genomic analysis showing that many saccharolytic *Bacteroidetes* including human gut *Bacteroides* as well as ruminant *Prevotella* spp., are equipped with a starch “sequestration” and enzymatic degradation systems encoded by *sus* (starch utilization system) gene clusters (Xu et al., 2003, 2007; Martens et al., 2009). In a recent study, an amylolytic system was identified in the genome of *R. bromii* that appears to be organized in a starch degrading enzyme complex referred to as “amylosome” (Ze et al., 2015).

After 4 h of incubation, *Allobaculum* spp. and other bacterial groups increased in relative abundance in the “heavy” gradient fractions. *Allobaculum* was shown to consume mono- and disaccharides, but not starch for growth (Greetham et al., 2004). Thus, we hypothesize that in our system RS was hydrolyzed into mono- and disaccharides within the first 2 h by the enzymatic machinery of primary degraders, i.e., mostly *Bacteroidetes*. After 4 h of incubation, other secondary RS degrading bacteria, e.g., *Allobaculum* spp., were able to benefit from the increased availability of the RS-derived sugars and metabolites. This hypothesis is further supported by a very recent RNA-SIP experiment by our group using *in vitro* incubation of murine fecal slurries with [U^13^C]glucose, in which *Allobaculum* spp. were identified as the most efficient glucose assimilators (Herrmann et al., 2017b).

No marked changes in the concentration or proportions of the measured fermentation products were observed during the course of the experiments. The complete lack of lactate following addition of RS is in agreement with previous RNA-SIP studies showing no production of lactate upon [U^13^C]starch fermentation (Kovatcheva-Datchary et al., 2009). Propionate and butyrate seem to have increased slightly upon addition of RS. This would be in line with the observed changes in the microbial populations, i.e., an increase in *Bacteroidetes* (in our data set represented by unclassified *Porphyromonadaceae*) and the high abundance of members of the *Lachnospiraceae* family (*Clostridium* cluster XIVa) in all samples of this study. These organisms have previously been linked to formation of propionate and butyrate (Macfarlane and Macfarlane, 2003; Cotta and Foster, 2006; den Besten et al., 2013; Yang et al., 2013; Salonen et al., 2014). However, the overall low levels of ^13^C-labeled SCFA, the limited number of biological replicates, and a considerable technical variability in our measurements do not allow to draw strong mechanistic conclusions and further experiments are required. Nevertheless, we were able to consistently detect ^13^C-labeled SCFA at levels above those expected to occur due to naturally present ^13^C. This suggests that some of the [U^13^C] has been assimilated and fermented by the bacterial community. In a previous study by our group it was also shown that [U^13^C]glucose was fermented within 2 h with most of the label being recovered from lactate, acetate, propionate and butyrate (Herrmann et al., 2017b). The isotopic data obtained here showed that the ^13^C-content of the investigated metabolites was rather low, but continually increased during the course of incubation. The profound difference in label incorporation rates of RS relative to glucose indicates a slower assimilation of the [U^13^C]starch. A slower fermentation of potato starch compared with corn starch was also observed in a rat cecal microbiota (Morita et al., 2013) and *in vitro* with a pig cecal microbiota (Martin et al., 1998). Altogether, these results indicate that degradation and utilization of (labeled) RS was taking place in our *in vitro* system, albeit at very slow rates. So far, it is unclear why fermentation rates of potato starch are rather low. However, it has been suggested that the big granule size of the potato starch in relation to other types of starch, results in a more limited surface area for enzymes to attach (Tester et al., 2006; Rocha et al., 2010). To substantiate the hypothesis of a slow fermentation, further experiments with longer incubation times and measurements to quantify (remaining) [U^13^C]starch in the samples at different time points are needed. Additionally, incorporation of the label into metabolites not covered in this analysis, such as gases (carbon dioxide, methane), other organic acids (succinate and formate), alcohols (ethanol and methanol) as well as into biomass needs to be investigated.

In conclusion, we demonstrated the suitability of RNA-SIP to unravel the phylogenetic identity of bacteria involved in carbohydrate fermentation within a complex intestinal community of murine origin. We have shown that the *Bacteroidetes*, in particular members of the *Prevotellaceae* and *Ruminococcaceae*, were highly active after starch addition, thereby largely corroborating previous studies. In future studies, the presented *in vitro* approach will be transferred to an *in vivo* feeding trial to investigate degradation of RS *in situ* in an intact ecosystem inside the host.

## Author contributions

EH, CR, and ME conceived and designed the study. EH performed the experiments. EH, WY, and DR contributed to the analysis of the data. All authors wrote the manuscript and approved the final version of the article.

### Conflict of interest statement

The authors declare that the research was conducted in the absence of any commercial or financial relationships that could be construed as a potential conflict of interest.
