# Supplementary material for: Determination of Resistant Starch Assimilating Bacteria in Fecal Samples of Mice by In vitro RNA-Based Stable Isotope Probing
Source: Front Microbiol. 2017 Jul 24;8:1331. doi: 10.3389/fmicb.2017.01331 (PMC5522855; doi:10.3389/fmicb.2017.01331)
Supplement: Supplementary file 1 [file Presentation1.PDF]

Supplementary Material

Determination of Resistant Starch Assimilating Bacteria in Fecal Samples of Mice by *in vitro* RNA-Based Stable Isotope Probing

Elena Herrmann[1], Wayne Young[2], Douglas Rosendale[3], Ralf Conrad[4], Christian U. Riedel[5*], and Markus Egert[1]

*** Correspondence:**
Christian Riedel
christian.riedel@uni-ulm.de

1 Supplementary Figures

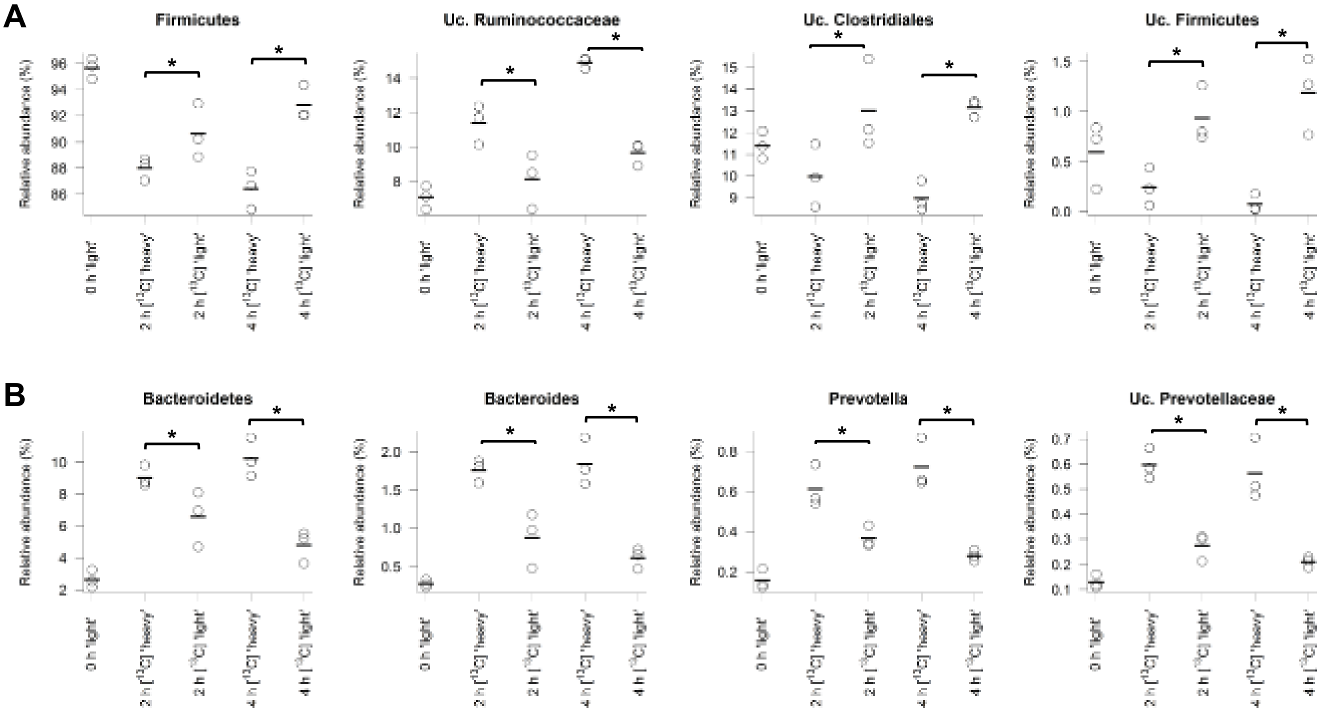

Supplementary Figure S1 | Relative abundance of selected bacterial taxa among the *Firmicutes* (**A**) and (**B**) *Bacteroidetes* represented by 16S rRNA gene amplicons in ‘light’ and ‘heavy’ RNA-SIP fractions. RNA was isolated from fresh fecal content (0 h) or after incubation with [^{13}C]starch for 2 and 4 h. Taxa with the most significant differences (FDR ≤ 0.006) in mean relative abundances between the ‘heavy’ and ‘light’ gradient fractions of the ^{13}C-labeled community are shown. Points indicate the percentage of total community (n = 3 fractions) and lines indicate the mean. * indicates two-factor permutation ANOVA significance in relative proportions at FDR ≤ 0.006 using time and density as factors.
